# High‐Voltage Rechargeable Aqueous Zinc‐Based Batteries: Latest Progress and Future Perspectives

**DOI:** 10.1002/smsc.202000066

**Published:** 2021-02-24

**Authors:** Yanxia Yu, Jinhao Xie, Haozhe Zhang, Ruofei Qin, Xiaoqing Liu, Xihong Lu

**Affiliations:** ^1^ MOE of the Key Laboratory of Bioinorganic and Synthetic Chemistry The Key Lab of Low-carbon Chem & Energy Conservation of Guangdong Province School of Chemistry Sun Yat-Sen University Guangzhou 510275 P. R. China

**Keywords:** aqueous electrolytes, decoupled battery systems, electrode design, high voltage rechargeable batteries, Zn-based batteries

## Abstract

Rechargeable aqueous zinc‐based batteries (AZBs) have been recently considered as desirable energy storage devices for renewable energy storage because of their high theoretical capacity, low cost, and high safety. Despite the inspiring achievements in this field, the energy density of AZBs is still far below expectation, which directly hinders their practical application as next‐generation secondary cells. To address this issue, previously, reseach efforts have been mainly dedicated to the improvement of capacity by designing different kinds of high‐capacity electrode candidates, especially the cathode materials. In recent years, elevation of the output voltage for energy density improvement has gained more and more attention. The main limitation of the relatively low output voltage of AZBs lies in the narrow electrochemically stable window of aqueous electrolytes, which results in limited choice of cathode materials with high potential. Herein, by summarizing recently reported work in this field, the strategies applied to high‐voltage AZB construction are classified into two categories, from the aspects of electrode and battery structure. Classic examples of each category are discussed in detail, and their respective advantages/defects are compared and commented on. Finally, further challenges are elaborated to provide more insights into this area.

## Graphical abstract

The energy density of the state‐of‐the‐art aqueous zinc‐based batteries (AZBs) is still far below expectation, which directly hinders their practical applications. The elevation of output voltage is demonstrated to be an effective strategy to address this issue. An overview of the latest development of the high‐voltage rechargeable aqueous zinc‐based batteries is presented. Further challenges and prospects are also discussed.

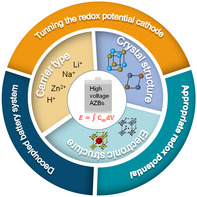

## 1 Introduction

The increasing requirement for green energy storage in large‐scale energy storage, electronic vehicles, consumer electronics, and other applications has promoted the exploration of state‐of‐the‐art energy storage technologies.^[^ ^1^ ^]^ Electrochemical storage system devices (ESDs) have emerged as one of the most attractive options for storing renewable energy, such as tidal, wind, and solar power.^[^ ^2^ ^]^ Recently, extensive attention have been paid to aqueous batteries due to the merits of high power density, safe operation, and inexpensive nature.^[^ ^3^ ^]^ Among recently reported aqueous batteries, rechargeable aqueous zinc‐based batteries (AZBs) have attracted great interest due to the following advantages of metallic zinc: 1) the high theoretical capacity (≈820 mA h g^−1^) and theoretical volume capacity (5854 mA h cm^−3^); 2) the suitable standard redox potential of Zn/Zn^2+^ (−0.76 V vs the standard hydrogen electrode (SHE)) and being chemically stable in air; 3) the relatively lower polarizability compared with other metals (e.g., Mg and Al); and 4) the high natural abundance.^[^ ^4^, ^5^, ^6^ ^]^ Moreover, the electrolytes used in AZBs are usually cheap, such as ZnCl_2_ and ZnSO_4_.^[^ ^7^ ^]^ In addition, compared with organic electrolytes, the ion conductivity of aqueous ones is substantially higher (about two orders of magnitude emhancement). All these excellent characteristics enable AZBs to function as a desirable option for large‐scale energy storage. Yet, the main obstacle hindering the practical application of AZBs is the energy density.^[^ ^8^ ^]^ Equation (1) shows the relationship between energy density and output voltage as well as discharge capacity.

(1) $$E=\int C_{m}dV$$

According to Equation (1), the energy density is the product of the discharge capacity and average output voltage. That is, the high energy densities (*E*, W h kg^−1^) of AZBs can be realized by increasing the specific capacity (*C*_m_, mA h g^−1^) and output voltage (*V*). As a key and active field, how to increase the energy stored in AZBs has been extensively explored, focusing primarily on specific capacity improvement.^[^ ^4^ ^]^ Many electrode materials for AZBs have been demonstrated to be suitable cathode candidates with high specific capacity for AZBs (e.g., layered V_2_O_5_ materials, α‐MoO_3_, and γ‐MnO_2_).^[^ ^9^ ^]^ Generally, the output voltage of AZBs is decided by the potential difference of redox reactions between the cathode and anode, and the cathode is the bottleneck for enhancing the output voltage of AZBs. Great efforts have been made to develop cathode materials with high electrode potential, including introducing transition‐metal‐based dopants or small molecules (e.g., Co^2+^, H_2_O). However, the low electrochemical stability window (ESW) of 1.23 V (potential difference between the hydrogen evolution reaction (HER) and oxygen evolution reaction (OER)) and limited electrode materials with redox potentials within the H_2_ and O_2_ evolution potentials results in a rather low output voltage of AZBs. A valid method to expand the ESW of an aqueous electrolyte is to use an alkaline electrolyte as the anolyte and an acidic electrolyte as the catholyte, in which the HER has a low potential in the alkaline electrolyte and the OER has a high potential in the acidic electrolyte. Following this approach, some redox couples with high electrode potential in an acidic electrolyte can be used to construct high‐voltage AZBs, such as MnO_2_/Mn^2+^ and PbO_2_/PbSO_4_. As the energy density is the product of the discharge capacity and average output voltage, searching for materials with a high output voltage platform can provide more space to improve the energy density. Currently, “high‐voltage” is still an ill‐defined concept. According to the state‐of‐the‐art literature, the definition of high‐voltage AZBs can be roughly divided into two cases. The first case is that high‐voltage batteries are mainly referred to as those possessing higher voltage than their counterparts. For example, Zn/Co_0.247_V_2_O_5_·0.944H_2_O batteries exhibit a high output voltage of nearly 1.7 V, higher than that of Zn/V_2_O_5_·*n*H_2_O, which is also known as a high‐voltage battery.^[^ ^10^ ^]^ The second case refers to those of which the output voltage is higher than that of rechargeable Zn//MnO_2_ batteries (1.2–1.4 V), one of the most promising aqueous battery systems for future commercilization. Despite the disparate definitions, in both cases, the output voltage of AZBs is dependent on different discharge conditions, especially for the current density. Generally, the output voltage of a battery usually undergoes a drop with the increasing of the current density due to its low electrolysis kinetics at high current density. However, up to now, there are few reviews that have comprehensively discussed and summarized the strategies concerning output voltage modifications of AZBs toward achieving high energy density.^[^ ^11^ ^]^

**Figure 1:**
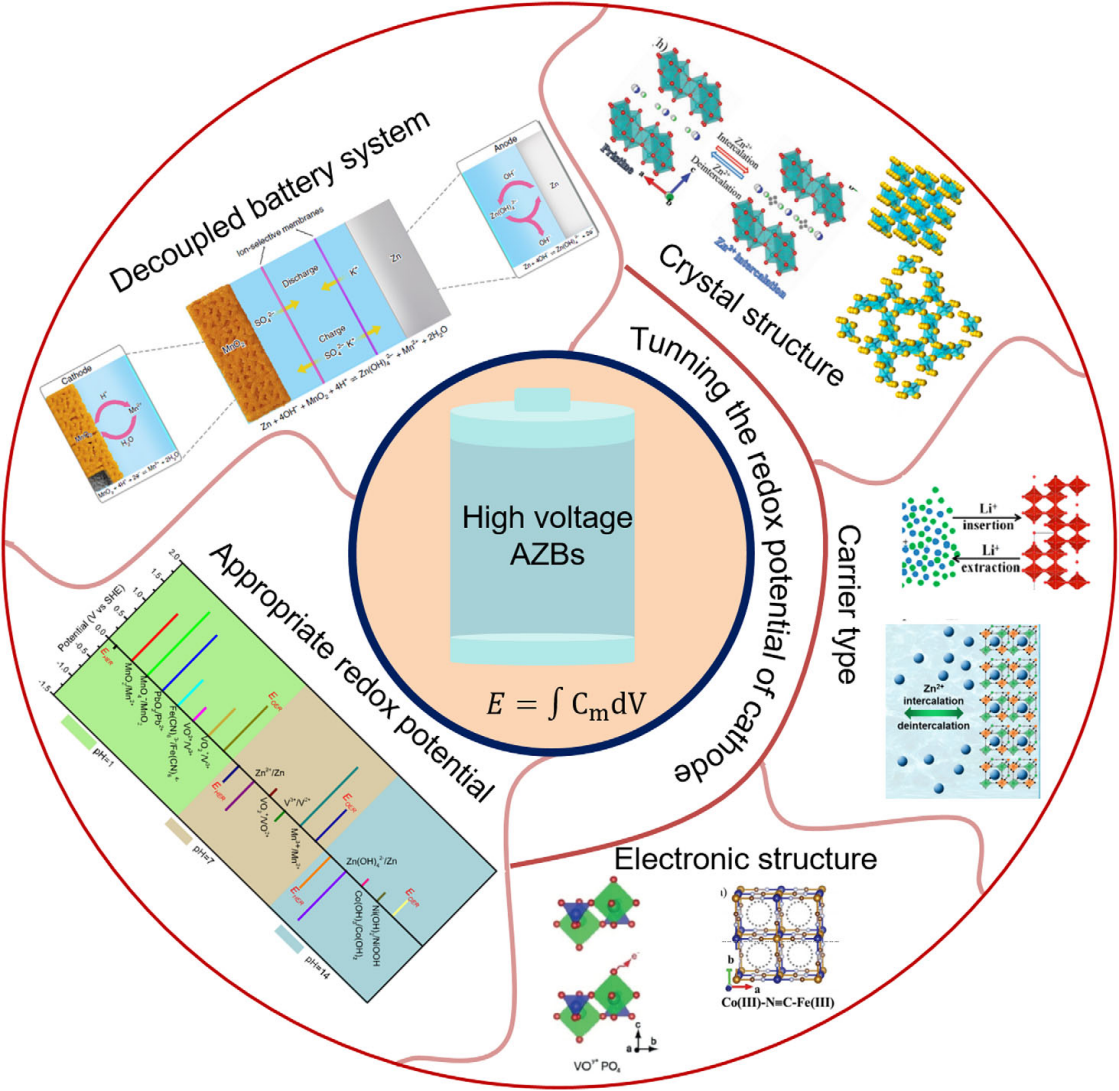
Schematic of strategies to improve the voltage of AZBs. Decoupled battery system. Reproduced with permission.^[^ ^77^ ^]^ Copyright 2020, Springer Nature. Crystal structure. (top image) Reproduced with permission.^[^ ^63^ ^]^ Copyright 2019, Wiley‐VCH. (bottom image) Reproduced with permission.^[^ ^113^ ^]^ Copyright 2019, American Chemical Society. Carrier type. (top image) Reproduced with permission.^[^ ^114^ ^]^ Copyright 2017, American Chemical Society. Electronic structure. (right image) Reproduced with permission.^[^ ^10^ ^]^ Copyright 2019, Wiley‐VCH. (left image) Reproduced with permission.^[^ ^65^ ^]^ Copyright 2019, Wiley‐VCH.

This minireview aims to provide clear design ideas for achieving high output voltage of AZBs. Special emphases are sequently put on discussing the challenge for constructing high‐voltage AZBs and summarizing the major design strategies for high‐voltage AZBs from the aspects of electrode and battery structure, including the selection of an appropritate redox potential of the cathode, tunning the redox potential of the cathode, and construction of a decoupled system (**Figure** **1**). Finally, remaining challenges and promising approaches in solving the voltage issue are presented for designing high‐voltage AZBs. We hope this review can shed light on promising directions for future research.

## 2 Issues Related to High‐Voltage AZBs

**Figure 2:**
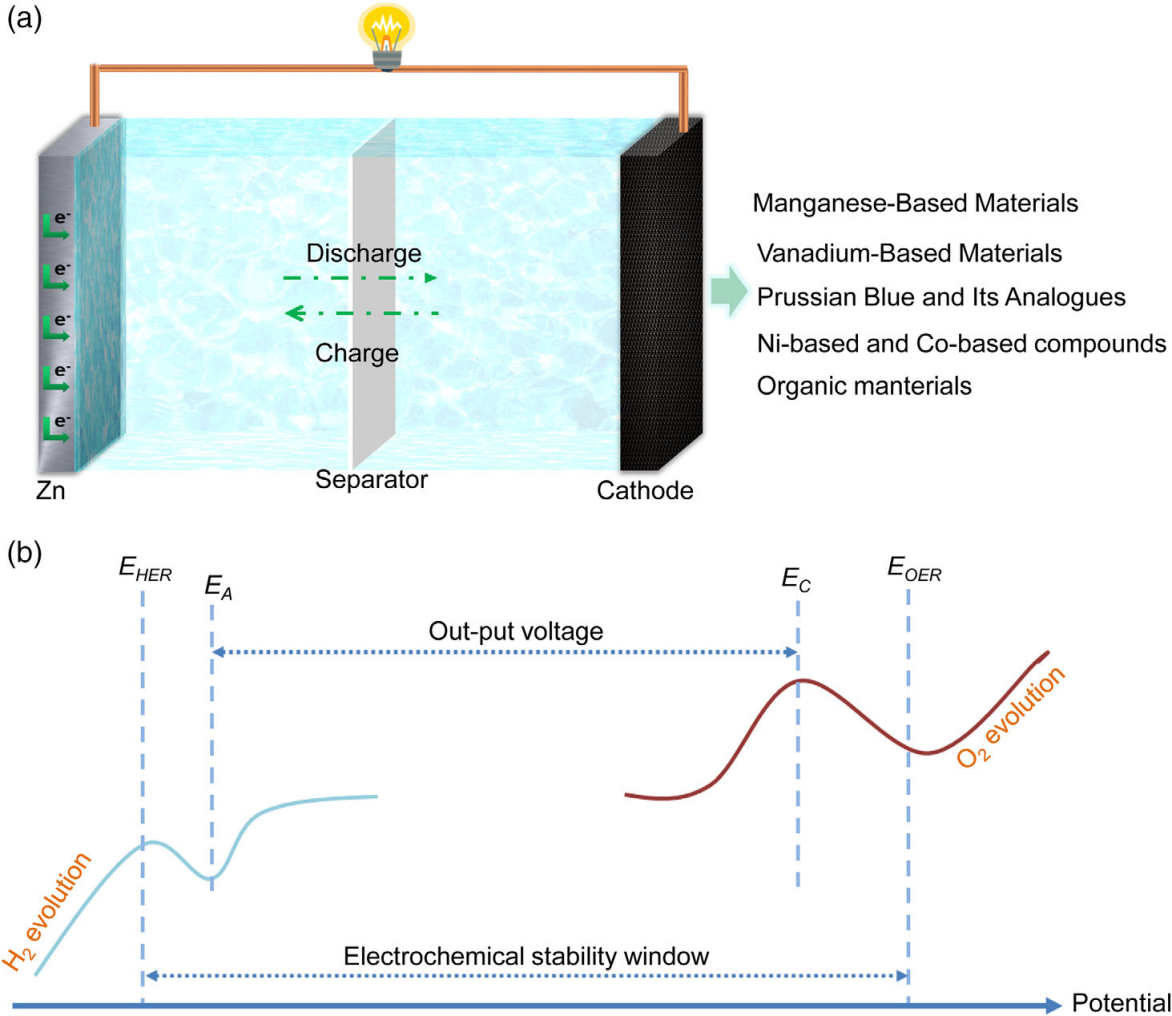
a) Illustration of the configuration of an AZB. b) A scheme illustration showing the output voltage of AZBs and ESW of an aqueous electrolyte.

Over the past 5 years, we have witnessed explosive progress on AZBs, but there are still some limitations that need to be solved. The configuration of AZBs is shown in **Figure** **2**a. The design of electrode materials compatible with the electrolyte plays an important role in achieving high‐energy‐density AZBs. Fundamentally, the ESW of water (≈1.23 V) is much lower than that of an organic electrolyte (>4 V),^[^ ^12^ ^]^ which strictly restricts the voltage output and confines the choice of electrode materials. Figure 2b displays a schematic illustration of the output voltage range of AZBs and the ESW of an aqueous electrolyte. The cathode is an oxidant and the anode is a reductant. The ESW of an electrolyte is determined by the lowest *E*_HER_ and highest *E*_OER_. In the ideal state, the prerequisite for obtaining a thermodynamically stable battery system is that the electrode electrochemical potentials (*E*_A_ for the anode and *E*_C_ for the cathode) are within the ESW of the electrolyte. In addition, when the potential applied on the battery is lower than *E*_HER_, or higher than *E*_OER_, electrolysis of water happens.

**Figure 3:**
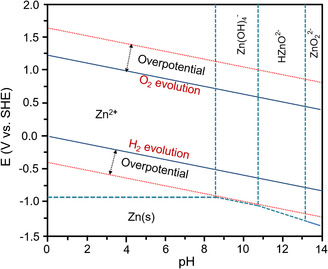
Pourbaix diagrams of a Zn–H_2_O system.

According to the Pourbaix diagram (**Figure** **3**), water decomposition is a pH‐dependent process (*E*_OER_ = 1.23 V − 0.059 pH V and *E*_HER_ = −0.059 pH V). However, simply regulating general pH does not alter the ESW of aqueous batteries because it only changes the potentials of the HER and OER instead of the potential difference of these two reactions. In neutral or acid aqueous electrolytes, the HER and OER are shown in Equation (2) and (3).

(2) $${2\text{ H}}^{+}+{2e}^{-}\to {2H}_{2}(0\text{ V}\;\text{vs}\;\text{SHE)}$$

(3) $${2H}_{2}O\;\to \;{4\text{ H}}^{+}\;+\;O_{2}\;+\;{4e}^{-}(1\text{.}23\text{ V}\;\text{vs}\;\text{SHE)}$$

In a neutral or alkaline aqueous solution, the water splitting reaction can be summarized in Equation (4) and (5).

(4) $${2H}_{2}O\;+\;2e^{-}\;\to \;2H_{2}\;+\;2{\text{OH}}^{-}(-0\text{.}83\;V\;\text{vs}\;\text{SHE)}$$

(5) $${2\text{OH}}^{-}\;\to \;{2H}_{2}O\;+\;O_{2}\;+\;4e^{-}(0\text{.}40\;V\;\text{vs}\;\text{SHE)}$$

In AZBs, the OER mainly occurs at the cathode side, while at the anode side, the HER takes place. The evolution of H_2_ and O_2_ not only consumes the electrolyte, but induces large polarization, leading to poor Coulombic efficiency (CE) and battery swelling.^[^ ^13^ ^]^ Therefore, how to extend the ESW of the electrolyte is a crucial issue that should be considered when designing materials for advanced AZBs with high energy density. Actually, the electrochemical reactions in an aqueous electrolyte are relatively complicated, and the actual water splitting often occurs at potentials higher than 1.23 V. The practical ESW of water could be presented as follows

(6) $$E_{\text{ESW}}=1.23+\varphi_{C}+\varphi_{A}+\varphi_{\text{other}}$$

where *φ*_A_ is the anodic overpotential, *φ*_C_ is the cathodic overpotential, and *φ*_other_ is the overpotential from other factors. In this case, designing and synthesizing electrode materials with high overpotential is an effective way to widen the ESW of the electrolyte. From this perspective, advanced catalyts for water decomposition should be avoided, such as S‐vacancy and edge‐activated 1T‐phase MoS_2_.^[^ ^14^ ^]^ In addition, the electrolytes, battery structure, etc. can work together to affect the overpotential of the electrode.^[^ ^15^, ^16^ ^]^ For example, the overpotential of the HER in neutral electrolytes could be readily up to 0.2 V (vs SHE), whereas the OER could expand to nearly 1.8 V (vs SHE).^[^ ^16^ ^]^

The type of electrolyte salt also affects the ESW of aqueous electrolytes. For example, Wessells et al. demonstrated that 1 m Li_2_SO_4_, 1 m NaNO_3_, 1 m LiNO_3_, and 1 m Mg(NO_3_)_2_ solutions deliver varied ESWs.^[^ ^17^ ^]^ Apart from this, increasing the concentration of electrolyte salts is another effective approach to expand the ESW of aqueous electrolytes. The increase of salt concentration is accompanied by the decrease of water content and the decline of water activity, resulting in enhanced potentials of the OER/HER. In addition, increasing the aqueous electrolyte concentration can alleviate the concentration polarization on the zinc surface, inhibiting the formation of Zn dendrites and decreasing the water number in the solvation sheath and free water molecules in the bulk electrolyte, thus preventing the corrosion of the Zn anode, and even dissolution of the cathode. It should be taken into account that the high concentration inevitably leads to decreased ionic conductivity due to the solvation behavior of cations. However, the hydration number and the radius of hydrated Zn^2+^ could be also affected by the concentration of the electrolyte. For example, the coordination number of Zn^2+^ with H_2_O would decrease and the interaction between Zn^2+^ and ${\text{SO}}_{4}^{2-}$ would be reinforced by increasing the concentration of a ZnSO_4_ electrolyte.^[^ ^18^ ^]^ In other words, the hydration number and hydrated cation radius are reduced in lower water surroundings, which is conducive to the rapid and reversible intercalation/deintercalation of hydrated Zn^2+^ ions into the cathode materials, thus enhancing the electrochemical performance of the battery. Therefore, the “water‐in‐salt” concept has been proposed in AZB systems. The exploit of the water‐in‐salt electrolyte (WISE) dates to 2015. Wang and co‐workers developed an ultrahighly concentrated electrolyte by dissolving 20 m bis(trifluoromethane sulfonyl)imide (LiTFSI) in water, which has a higher mass and volume of salt than water; thereby, it was termed as WISE.^[^ ^19^ ^]^ Interestingly, the ESW of this WISE could be expanded up to 3 V because the absence of free water made it more difficult for water decomposition. Following this work, many different types of concentrated electrolytes have been developed to broaden the ESW of electrolytes,^[^ ^20^ ^]^ which further changes the selection scope of electrode materials. Inspired by the“water‐in‐salt” concept, Xu and co‐workers developed a WISE, namely, 1 m Zn(TFSI)_2_ + 20 m LiTFSI, to improve the stability of the Zn anode.^[^ ^21^ ^]^ The Zn//LiMn_2_O_4_ battery based on this electrolyte delivers a high energy density of 180 W h kg^−1^ and output voltage of up to 1.8 V. Although the WISE can effectively widen the ESW of the aqueous electrolyte, it is still a challenge to design and synthesize suitable electrode materials to construct high‐voltage AZBs in a WISE. Recently, the addition of redox‐active additives into the electrolyte has been proved to be an effective way to widen the ESW of the electrolyte. These redox‐active additives need to meet the following criteria: 1) high solubility in water and 2) the redox reaction should occur on at least one surface of the electrodes (cathode and anode). To this end, Br^−^/${\text{Br}}_{3}^{-}$, I^−^/$I_{3}^{-}$, and [Fe(CN)_6_]^4−^/[Fe(CN)_6_]^3−^ are promising redox species to extend the ESW of the aqueous electrolyte.

Metallic Zn is one of the most widely used anode materials for AZBs, but it suffers from dendrite formation and low CE of Zn^2+^ electroplating/stripping.^[^ ^22^ ^]^ According to the Pourbaix diagram of the Zn/H_2_O system, the redox potential of metallic Zn depends on the pH values, and a lower redox potential can be achieved in an alkaline solution. However, in alkaline electrolytes, $\text{Zn}{\left(\text{OH}\right)}_{4}^{2-}$ is the charge carrier, and a high concentration of $\text{Zn}{\left(\text{OH}\right)}_{4}^{2-}$ causes the irreversible formation of insulating ZnO or Zn(OH)_2_, giving rise to the passivation of Zn anodes. These insulating ZnO precipitates are difficult to reduce to Zn, which results in poor rechargeability. Consequently, the AZBs exhibit serious capacity fading and decreased output voltage upon cycling. In addition, the formation of Zn dendrites imposes more serious issues such as severe polarization and unsatisfactory CE.

Structural transition of cathode electrodes during the cycling process is another issue facing high‐voltage AZBs. For example, manganese dioxide (MnO_2_) has a lot of phases, such as α‐, β‐, γ‐, and δ‐MnO_2_, and has been proved to be an ideal host material for Zn^2+^ storage in an aqueous electrolyte.^[^ ^23^ ^]^ However, the MnO_2_ hosts suffer from complicated phase transitions during the cycling process, which bring out a series of undesirable consequences, such as huge volume changes and irreversible structural changes, thus leading to blockages of the Zn^2+^ diffusion path.^[^ ^24^ ^]^ Beyond that, the volume change could result in an unstable interface between the electrode and electrolyte that severely interferes with repeated ion insertion/extraction, which may lead to increased polarization and thus lower the output voltage of the battery.

## 3 Toward High‐Voltage AZBs: Strategies and Advancements

In most cases, the practical output voltage of AZBs cannot fully agree with the theoretical voltage range. Generally, AZBs mainly consist of three parts: the electrode (cathode and anode), electrolyte (solute and solvent), and separator. The output voltage of AZBs is related to the all these components, but the eletrode substance is generally considered to play a decisive role. In this part, we classify the modification strategies for voltage elevation of AZBs, which have been reported to be effective in recent works, into three major categories. These strategies involve the selection of an appropriate redox potential of the cathode, redox potential tuning of the cathode to fully make use of the ESW of aqueous electrolytes, and design of decoupled systems. By adopting effective strategies and an appropriate battery system, the voltage of AZBs can be increased to 2.5 V or even higher.

### 3.1 Selection of Appropriate Redox Potential of Electrode

Generally, the output voltage of AZBs (*V*) is mainly determined by the difference in electrode potentials between the cathode and the anode (denoted as *E*_C_ and *E*_A_, respectively), as shown in Equation (7).

(7) $$V\;=\;E_{C}-E_{A}$$

The electrode potential of the redox reaction on an electrode can be calculated by the Nernst equation

(8) $$E=E^{\theta }+\frac{2.303\;RT}{nF}\mathrm{lg}\frac{{\left(\text{Ox}\right)}^{m}}{{\left(\text{Red}\right)}^{q}}$$

**Figure 4:**
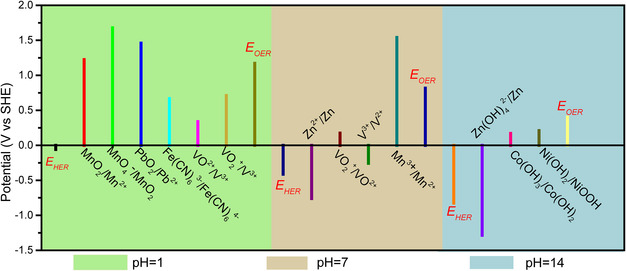
Standard potentials of Zn, V‐based, Mn‐based, Pb‐based, Co‐based, and Ni‐based compounds.

Equation (8) describes the relationship between the electrode potential and system composition, where *E* ^*θ*^ represents the standard electrode potential and *R*, *T*, *F*, *K*, (Ox), and (Red) are the gas constant, temperature, Faraday constant, equilibrium constant of water ionization, oxidizing state activity, and reducing state activity. The stronger the oxidation ability of the cathode is, the more positive is the electrode potential. On the contrary, the stronger the reduction ability of the anode is, the more negative is the electrode potential. Theoretically, the maximum output voltage can be easily obtained by choosing a strong oxidizer as cathode and strong reducing agent as anode. Yet, for AZBs, the choice of anode is almost limited to the Zn metal, of which the electrode potential is fixed (−0.76 vs SHE in a neutral or acidic solution, 1.228 vs SHE in an alkaline solution). Thus, the key to constructing high‐voltage AZBs is to choose a suitable cathode material that has a lower electrode potential than Zn metal. **Figure** **4** visualizes the standard potentials of redox couples in some reported cathode materials. The basic requirements for selecting cathode materials is that the electrode potentials should be within the ESW of the electrolyte to ensure the feasibility of the electrodes. It should be pointed out that V‐based materials have aroused widespread interest due to their relatively high theoretical capacity (e g., the capacity of V_2_O_5_ is ≈400 mAh g^−1^) and high energy density of 250 Wh kg^−1^.^[^ ^25^ ^]^ However, for Prussian blue analogues (PBAs), despite their low storage capacity for Zn^2+^ (<140 mAh g^−1^), they could also exhibit an energy density up to 232 Wh kg^−1^, which is mainly attributed to their high output voltage (≈1.7 V vs Zn/Zn^2+^).^[^ ^26^ ^]^

**Figure 5:**
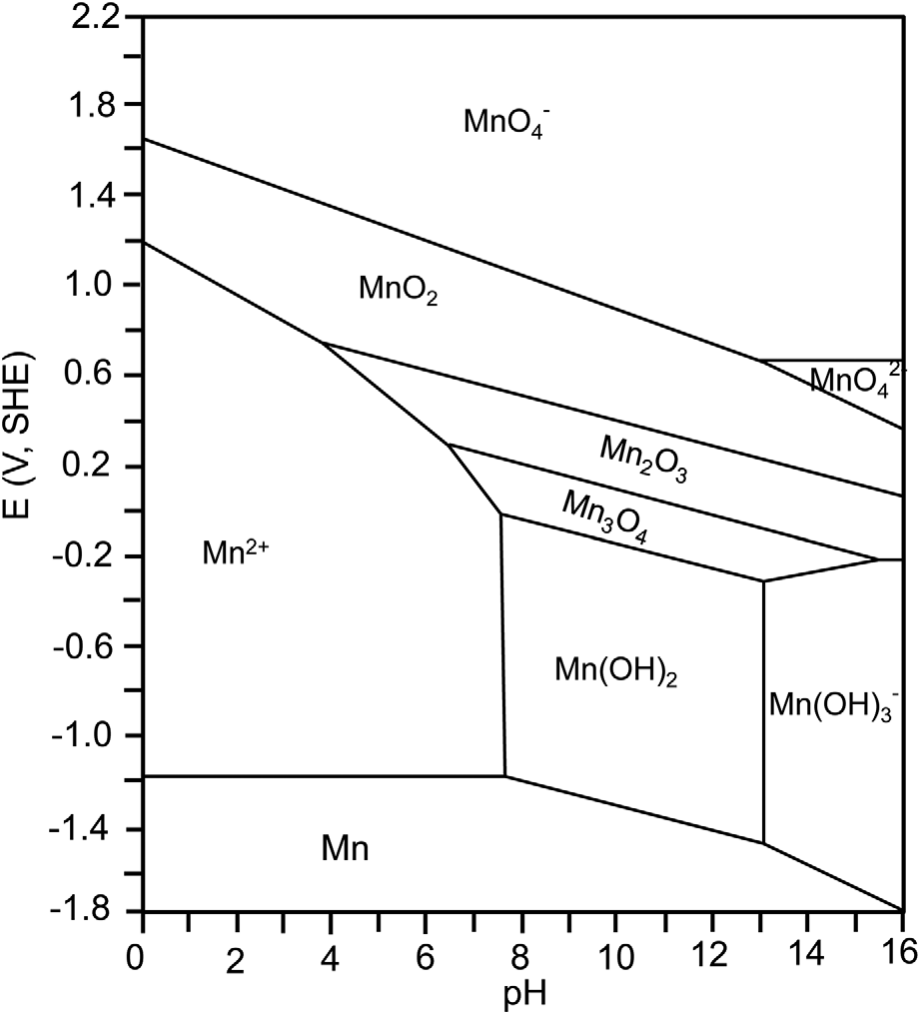
Pourbaix diagram of a Mn–H_2_O system. Reproduced with permission.^[^ ^78^ ^]^ Copyright 2020, Wiley‐VCH.

Manganese (Mn)‐based materials are also promising high‐voltage cathodes for AZBs in consideration of the suitable redox potential of Mn. As shown in the Pourbaix diagram (**Figure** **5**), MnO_2_, in an alkaline solution, is reduced to Mn(OH)_2_, Mn_3_O_4_, and Mn_2_O_3_ at low voltage, which exhibit low redox potential. For instance, alkaline Zn//MnO_2_ batteries deliver an output voltage that is below 1.5 V (vs Zn/Zn^2+^),^[^ ^27^ ^]^ which severely limites their further application in large‐scale energy storage systems (ESS). The alkaline condition makes the zinc anode more stable, but it decreases the MnO_2_ performance due to the formation of irreversible intermediates such as Mn_2_O_3_, Mn_3_O_4_, and Mn(OH)_2_.^[^ ^27^, ^28^ ^]^ The recently developed rechargeable Zn//MnO_2_ batteries based on neutral or slightly acidic electrolytes have aroused much attention due to their low cost, moderate discharge potentials, and relatively high theoretical capacity up to nearly 308 mA h g^−1^.^[^ ^29^, ^30^ ^]^ In this case, the most widely accepted mechanism is the cationic redox, which refers to the reduction/oxidation of Mn^4+^ ↔ Mn^3+^ ↔ Mn^2+^ during the reversible insertion/extraction of Zn^2+^ ions. During the discharge process, Zn^2+^ ions are intercalated into the MnO_2_ structure, together with the partial reduction of Mn^4+^ to Mn^3+^. Then, the unstable Mn^3+^ ions tend to disproportionate into Mn^4+^ and Mn^2+^, which could dissolve into the electrolyte. Upon charging, the dissolved Mn^2+^ ions are electrochemically oxidized to MnO_2_. Thus, the key redox in the applied cathode is the Mn^4+^/Mn^3+^, constraining their capacities below 308 mAh g^−1^ and output voltages below 1.4 V (vs Zn/Zn^2+^). However, due to the presence of H^+^ in mildly acidic Zn^2+^‐based electrolytes, the two‐step H^+^ and Zn^2+^ insertion/extraction also has been identified as a feasible mechanism, which was first proposed by Wang and co‐workers.^[^ ^31^ ^]^ In their work, two distinct plateaus were observed during the discharge process from the galvanostatic intermittent titration technique (GITT) profiles of the Zn/α‐MnO_2_ battery, indicating the insertion of two different kinds of ions. Note that the insertion of H^+^ exhibits a higher output voltage than that of Zn^2+^ due to its fast diffusion rate. Very recently, Mai and co‐workers found that reversible deposition/dissolution of MnO_2_/Mn^2+^ could be realized by the addition of manganese sulfate additive, which could effectively improve the stability of Zn/MnO_2_ batteries.^[^ ^32^ ^]^ Note that the multivalency of Mn gives it a significant potential for two‐electron Mn^4+^/Mn^2+^ reactions at a high redox potential of 1.22 V (vs SHE).^[^ ^33^ ^]^ According to the Pourbaix diagrams of the Mn–H_2_O system in Figure 5, the Mn^2+^/MnO_2_ cathode in the acid solution effectively postpones the potentials for the OER and exhibits high redox potentials. For instance, Chao et al. revealed that adding 0.1 m H_2_SO_4_ to a solution containing Mn^2+^ ions could effectively realize the redox reaction of MnO_2_/Mn^2+^ in a Zn//MnO_2_ battery.^[^ ^34^ ^]^ The electrochemical reactions in the cathode and the electrode potential can be summarized as follows

(9) $${\text{Mn}}^{2+}\;+\;{2H}_{2}O↔{\text{MnO}}_{2}+{4\text{ H}}^{+}+{2e}^{-}E_{0}=1\text{.}228\;\text{vs}\;\text{SHE}$$

Along this line, many other transition‐metal‐derived compounds, such as nickel (Ni)‐based and cobalt (Co)‐based compounds, have also been developed for the cathode for AZBs due to their high electrochemical activity and highly reversible redox reaction, including Ni(OH)_2_,^[^ ^35^ ^]^ Ni/NiO,^[^ ^36^ ^]^ NiCo_2_O_4_,^[^ ^37^, ^38^ ^]^ Co_3_O_4_,^[^ ^39^, ^40^ ^]^ and Co_3_O_4_@NiO.^[^ ^40^ ^]^ These Ni‐ or Co‐based oxide materials in an alkaline electrolyte can react with OH^−^ to store charges. Taking Co_3_O_4_ as an example, the reaction mechanism can be described as

(10) $${\text{Co}}_{3}O_{4}+{\text{OH}}^{-}↔3\text{CoOOH}\;+\;e^{-}$$

(11) $$3\text{CoOOH}+3{\text{OH}}^{-}↔3{\text{CoO}}_{2}+3H_{2}O+3e^{-}$$

During the charging process, Co_3_O_4_ and OH^−^ undergo a two‐step reaction to convert CoO_2_ from the alkaline electrolyte.^[^ ^39^ ^]^ Upon discharging, the CoO_2_ is reduced to Co_3_O_4_. In the redox reaction between the OH^−^ ion and Co_3_O_4_, the redox peaks are located at 0.464/0.415 and 0.37/0.286 V, respectively. Thus, it should be pointed out that Ni‐ or Co‐based materials that operate in an alkaline electrolyte exhibit a high output voltage, which is mainly attributed to the fact that Zn has a lower redox potential in alkaline solution (−1.22 vs SHE).^[^ ^38^ ^]^ For example, in an alkaline electrolyte, a Ni//Zn battery based on a Ni–NiO cathode delivers an output voltage of around 1.75 V (vs Zn/Zn^2+^) at different current densities.^[^ ^36^ ^]^ In addition, iodine has a relatively high redox potential (I_2_/I^−^, 0.536 V vs SHE) and high theoretical capacity (211 mAh g^−1^, based on the mass of iodine), and has also been proved to be another suitable cathode with Zn metal. Typically, aqueous Zn//I_2_ batteries could deliver a high output voltage of above 1.2 V (vs Zn/Zn^2+^).^[^ ^41^, ^42^, ^43^, ^44^ ^]^

Compared to inorganic compounds, organic materials are promising candidates for the cathode in AZBs due to their merits of light weight, metal‐free nature, and flexibility. Notably, the structural diversity and functionalization of organic materials subtly control their redox potentials, thus providing many possibilities for improving the cell voltage. Organic carbonyl compounds and conductive polymers are typical representatives of organic cathode materials for AZBs. For instance, calix[4]quinone (C4Q) was designed to act as the cathode material for AZBs due to its open bowl structure and eight carbonyls.^[^ ^45^ ^]^ The redox center for Zn^2+^ ion storage in C4Q is determined by the carbonyl group with more negative electrostatic potential. On discharging, the Zn^2+^ ions could be stored by coordinating to the oxygen atoms in the electrochemically reduced carbonyl groups, and the Zn^2+^ ions are removed after the charge process. As a result, the AZBs based on C4Q cathode provided a flat output voltage of 1.0 V (vs Zn/Zn^2+^). After that, a variaty of organic cathodes coupled with carbonyl groups were reported, such as tetrachloro‐1,4‐benzoquinone,^[^ ^46^ ^]^ pyrene‐4,5,9,10‐tetraone,^[^ ^47^ ^]^ and 3,4,9,10‐perylenetetracarboxylic dianhydride.^[^ ^48^ ^]^ However, the redox potentials of these organic cathodes is usually between 0.24 and 0.34 V. Compared with organic carbonyl materials, conductive polymers with long‐range π‐conjugative structure have higher structural stability and electrical conductivity. However, the discharge platforms of most conductive polymers reported so far are not obvious,^[^ ^49^, ^50^ ^]^ and they all show an inclined discharge curve, with the redox potential only around 0.24 V (vs SHE), which involves their complicated ion storage mechanism. Taking polyaniline (PANI) as an example, Zn^2+^ insertion/extraction and dual‐ion mechanisms were developed for Zn//PANI batteries.^[^ ^51^ ^]^ Upon first discharge, the doped nitrogen (=NH^+^ groups) are reduced to —NH—, accompanied by the removal of Cl^−^. At the same time, the undoped (=N—) nitrogen is to —N^−^—, which could react with Zn^2+^ ions. At the following charge process, the stored Zn^2+^ ions will be released together with the oxidation of —N^−^— to =N—. In addition, —NH— will be oxidized to the doped state (=NH^+^—) to attract CF_3_ ${\text{SO}}_{3}^{-}$. In subsequent cycles, fully oxidized PANI is first reduced to half‐oxidized PANI, along with the release of CF_3_ ${\text{SO}}_{3}^{-}$. Then from half‐oxidized PANI to fully reduced PANI, CF_3_ ${\text{SO}}_{3}^{-}$ is released from PANI continuously and Zn^2+^ interacts with PANI simultaneously. The following charging process involves the release of Zn^2+^ ions and the ingestion of CF_3_ ${\text{SO}}_{3}^{-}$ out/into PANI, respectively.

### 3.2 Tuning the Redox Potential of the Cathode

#### 3.2.1 Regulation of Crystal Structure

**Figure 6:**
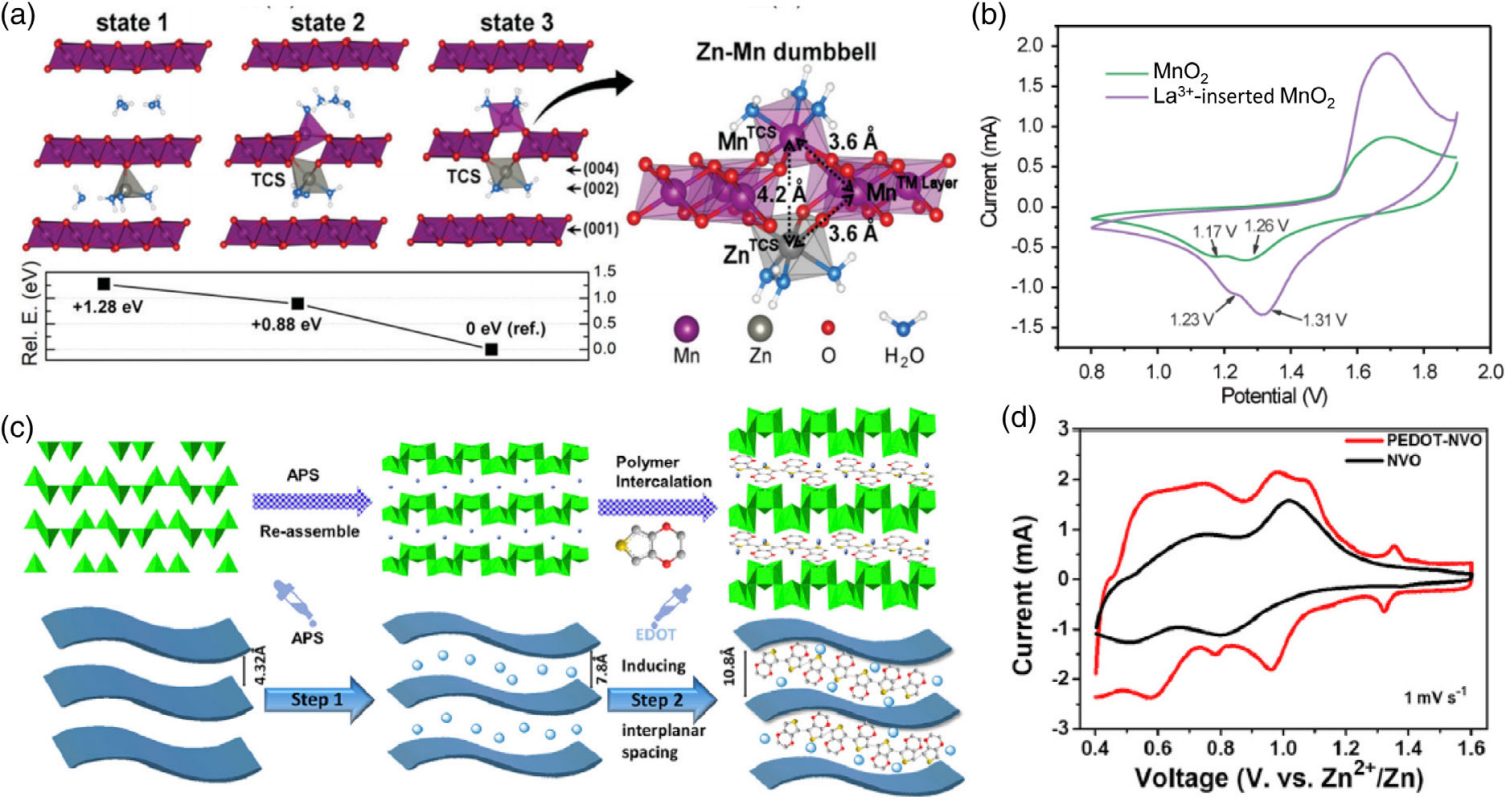
a) Illustration of Zn^2+^ and crystal water–intercalated structures and relative energies (left panel). Reproduced with permission.^[^ ^55^ ^]^ Copyright 2019, Royal Society of Chemistry. b) CV curves of MnO_2_ and La^3+^‐inserted MnO_2_ at 1 mV s^−1^. Reproduced with permission.^[^ ^56^ ^]^ Copyright 2019, Royal Society of Chemistry. c) Schematic diagram of preparation process of PEDOT‐intercalated NVO layered materials and d) CV curves of NVO and PEDOT‐intercalated NVO at 0.1 mV s^−1^. c,d) Reproduced with permission.^[^ ^57^ ^]^ Copyright 2020, Elsevier Inc.

As mentioned before, more and more cathode materials with suitable electrode potential have been developed, including Mn‐based and V‐based materials, Prussian blue compounds, and organic materials. However, most reported cathode materials cannot reach their theoretical electrode potential. The main reason is that the strong electrostatic interaction between Zn^2+^ and host frameworks makes Zn^2+^ diffusion sluggish, which affects the reversibility of the battery. As is well known, MnO_2_ is the most ideal candidate for Zn^2+^ storage because of its tunnel or layered structure that is favorable for the reversible insertion/deinsertion of Zn^2+^. Importantly, MnO_2_ has different crystallographic polymorphs, including α‐, β‐, γ‐, R‐type (tunnel structures), δ‐ (layered structure), and spinel‐type MnO_2_. The crystal structure of MnO_2_ has an important effect on the voltage characteristics of AZBs. For instance, the tunnel‐type MnO_2_ polymorphs, such as α‐MnO_2_ and γ‐MnO_2_, have similar output voltage of around 1.4 and 1.3 V (vs Zn/Zn^2+^),^[^ ^52^, ^53^ ^]^ and layered δ‐MnO_2_ has a lower output voltage of 1.23 V.^[^ ^54^ ^]^ In addition, the output voltage of layered MnO_2_ could be improved by incorporating small molecules into the layered structure. For example, Choi and co‐workers realized a high voltage of 1.55 V by incorporating crystal water into layered MnO_2_, as shown in **Figure** **6**a.^[^ ^55^ ^]^ In this work, the existence of interlayer crystal water not only effectively facilitates Zn^2+^ diffusion by decreasing the electrostatic interactions between Zn^2+^ ions and the host structure, but also suppresses the side reactions during electrochemical cycling. As a consequence, this Zn//MnO_2_ battery delivers an output voltage up to 1.55 V (vs Zn/Zn^2+^), which is higher than that of most reported Zn–MnO_2_ batteries.

In addition to water molecules, some metal ions with a large hydrated radius can also be preinserted into the cathode material to improve the reversible insertion/extraction capability of Zn^2+^. These intercalation materials have the following advantages: 1) The intercalated large size metal ion could stabilize the host structure and widen the interlayer spacing and 2) the electrostatic interactions between the host structure and the inserted Zn^2+^ can be effectively decreased due to the charge shielding effect. Monovalent alkali metal ions (e.g., Li^+^, Na^+^, and K^+^) and multivalent metal ions (e.g., Ca^2+^, Mg^2+^, and La^3+^) are considered the most promising intercalated cations for layered cathodes. For example, La^3+^‐intercalated δ‐MnO_2_ was obtained as the cathode for AZBs.^[^ ^56^ ^]^ As shown in Figure 6b, the overpotential gap of the redox peaks for La^3+^‐intercalated MnO_2_ is smaller than for prisine MnO_2_ due to its reversible Zn^2+^ insertion and extraction. Therefore, the aqueous Zn‐ion battery based on the La^3+^‐intercalated MnO_2_ cathode delivered a higher output voltage than prisine MnO_2_.

Another notable thing is that conjugated conductive polymers,such as poly(3,4‐ethylenedioxythiophene) (PEDOT), polyaniline, and polypyrrole, can also be used as guest species to enhance the interlayer spacing of the layered cathode materials. As shown in Figure 6c, PEDOT‐intercalated NH_4_V_3_O_8_.0.5H_2_O (NVO) was obtained by the redox‐intercalative polymerization of 3,4‐ethylenedioxythiophene monomers.^[^ ^57^ ^]^ The interplanar spacing of NVO was enlarged from 0.78 to 1.08 nm by the introduction of PEDOT. In addition, an oxygen vacancy was formed with the assistance of the polymerization and insertion of the EDOT monomer, giving rise to enhanced charge transport in the PEDOT‐intercalated NVO. The reduction/oxidation of V in PEDOT‐intercalated NVO material refers to V^5+^ to V^3+^ rather than V^4+^ to V^3+^ during the reversible insertion/extraction of Zn^2+^ ions. Accordingly, AZBs based on a PEDOT‐intercalated NVO cathode exhibit a capacity of 356.8 mAh g^−1^ at 0.05 A g^−1^ and excellent capacity retention (94.1% after 5000 cycles at 10 A g^−1^). Moreover, the PEDOT–NVO electrode has lower polarization than NVO (Figure 6d), demonstrating its highly reversible (de)intercalation courses. As a consequence, the PEDOT–NVO cathode delivers an output voltage up to 1.0 V (vs Zn/Zn^2+^), which is higher than that of NVO (0.9 V).

#### 3.2.2 Regulation of Electronic Structure

Poor electrical conductivity of materials is not conducive to the electron transfer process. Defect engineering, such as vacancy generation and heteroatom doping, is a well‐established strategy to regulate the electronic structure of materials and thereby affects the adsorption energy of zinc ions.^[^ ^50^ ^]^ In addition, defect engineering could bring other advantages: 1) increasing the specific surface area to facilitate ion and electron transfer and 2) enhancing the Fermi energy level or lowering the bandgaps of materials to increase the electrical conductivity. Thus, materials with metal ion doping have been extensively investigated to improve the electrochemical performance of AZBs, such as V^5+^‐doped MnO_2_ nonoparticles,^[^ ^58^ ^]^ Ag‐doped V_2_O_5_,^[^ ^59^ ^]^ and Al‐doped VO_1.52_(OH)_0.77_.^[^ ^60^ ^]^

**Figure 7:**
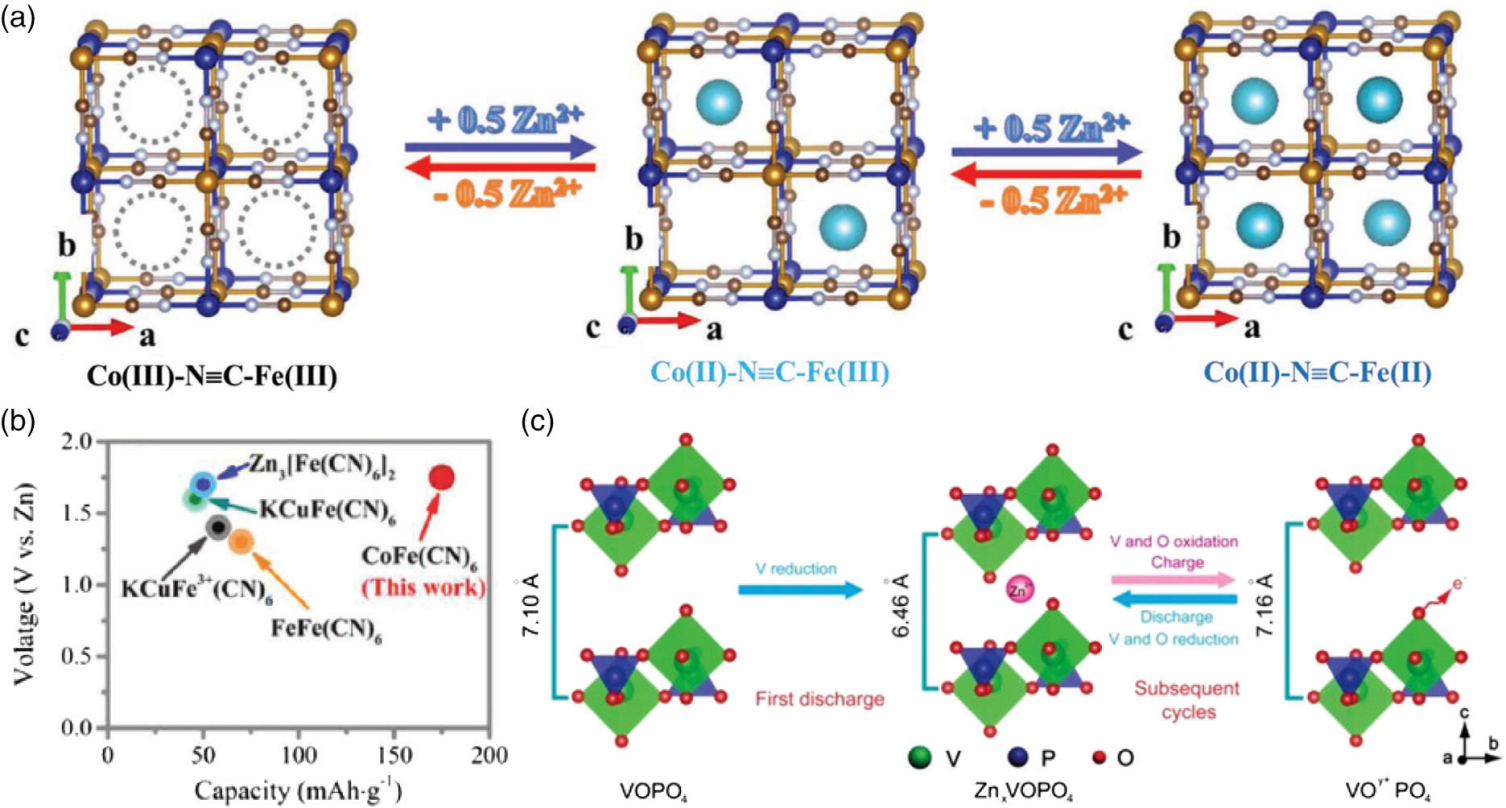
a) Schematic illustration of reversible Zn^2+^ insertion/extraction in CoFe(CN)_6_ frameworks during the cycling process. b) Capacity and voltage comparison of the CoFe(CN)_6_ electrode with reported PBA cathode for AZBs. a,b) Reproduced with permission.^[^ ^10^ ^]^ Copyright 2019, Wiley‐VCH. c) The redox mechanism of oxygen and vanadium in a VOP_4_ cathode. Reproduced with permission.^[^ ^65^ ^]^ Copyright 2019, Wiley‐VCH.

The pristine V‐based compounds suffer from a low output voltage because the two‐electron reaction exhibits relatively low redox potentials at 0.04–0.14 V (V^5+^/V^4+^) and −0.16 to 0.06 V (V^4+^/V^3+^) versus SHE. Doping metal ions, such as Zn^2+^, Ca^2+^, Ag^+^, and Li^+^, into V‐based compounds has been intensively studied, which delivers a feasible capacity (>300 mAh g^−1^).^[^ ^61^ ^]^ However, metal ion–doped V‐based compounds also display unsatisfactory output voltage (<1.0 V vs Zn/Zn^2+^).^[^ ^62^ ^]^ This might be attributed to the fact that these metal ions just act as a pillar to retain the structure of the materials during cycling, but do not change the electrical structure. Therefore, the choice of the doped metal ions plays an important role in improving the output voltage of AZBs. Recently, Zhi and co‐workers found that high‐voltage Zn//V batteries could be obtained by doping Co into V‐based oxides (**Figure** **7**a).^[^ ^63^ ^]^ As a result, a Zn//V battery based on a Co–V_2_O_5_ cathode provided 52.54% capacity (227 mAh g^−1^) above 1.0 V (vs Zn/Zn^2+^), whereas the output voltage of Zn//V_2_O_5_ is almost always lower than 1.0 V (vs Zn/Zn^2+^). The increased output voltage is mainly due to the high electrode potential of V^5+^/V^4+^, which is led by the increased interaction between the Co 3*d* and V 3*d* orbits. Similarly, the output voltage of PBAs also could be effectively increased by incorporating Co into PBA materials,^[^ ^10^ ^]^ which is attributed to the redox reaction of Co^3+^/Co^2+^ and Fe^3+^/Fe^2+^. Reversible Zn^2+^ intercalation/deintercalation from CoFe(CN)_6_ frameworks are shown in Figure 7b. CoFe(CN)_6_ exhibits a high output voltage of 1.75 V, which is higher than that of the Zn//FePBAs (≈60 mAh g^−1^, 1.2 V). The introduction of the anionic redox reaction could also enhance the electrode potentials of the host materials.^[^ ^64^ ^]^ For instance, the oxygen redox reaction can be activated by introducing the P—O covalence into the V_*x*_O_*y*_ polyhedra.^[^ ^65^ ^]^ For the Zn//VOP_4_ battery, except for the conventional redox reaction between V^5+^ and V^4+^, the redox process of lattice oxygen atoms in VOPO_4_ also takes place in the high‐voltage region (Figure 7c), thereby increasing the output voltage of Zn/VOPO_4_ batteries to ≈1.56 V.

### 3.3 Controlling the Species of Carriers

**Figure 8:**
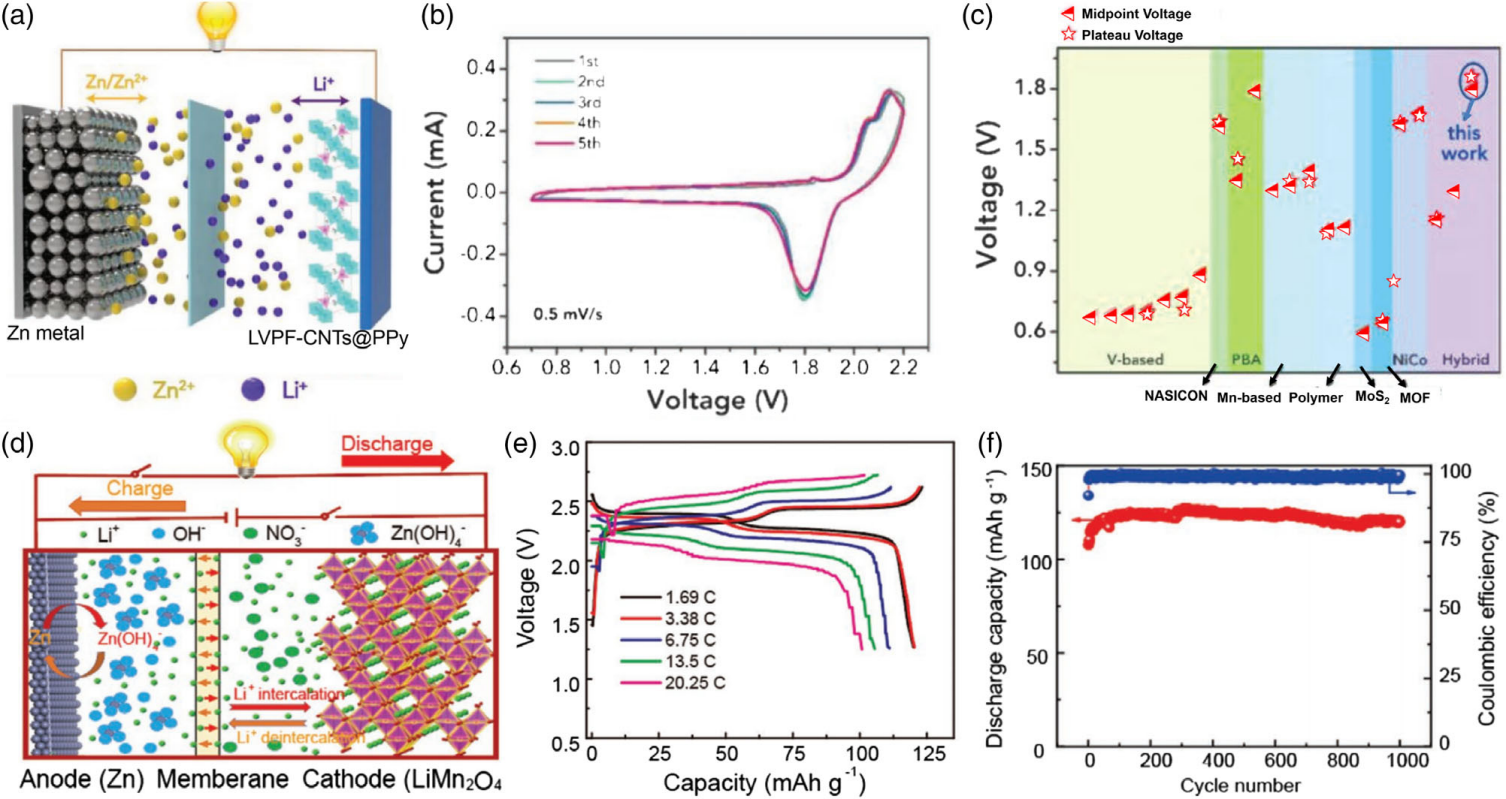
a) Scheme of the working mechanism of AZBs based on LVPF–carbon nanotube (CNTs)@polypyrrole(ppy) as cathode, b) CV curves of the AZBs, and c) comparision of output voltage of the Zn‐hybrid battery with recently reported zinc battery systems. a–c) Reproduced with permission.^[^ ^71^ ^]^ Copyright 2019, Wiley‐VCH. d) Schematic representation of the structure and working mechanism of the assembled zinc‐hybrid battery consisting of a Zn–LiMn_2_O_4_ electrode and LiOH–LiNO_3_ hybrid electrolyte, e) Voltage‐specific capacity profiles of the zinc‐hybrid battery at various current densities from 1.69 to 20.25 C and f) cycling performance. d–f) Reproduced with permission.^[^ ^72^ ^]^ Copyright 2020, Wiley‐VCH.

At present, the most widely accepted mechanism for most zinc ion battery cathodes is reversible Zn^2+^ insertion/extraction. In this case, Zn^2+^ ions act as carriers traveling back and forth between the anode and cathode during the charge/discharge process. Taking α‐MnO_2_ as an example, upon charging, Zn^2+^ ions are extracted from α‐MnO_2_. Upon discharging, Zn^2+^ ions will insert into the tunnels of α‐MnO_2_. However, the strong Coulomb repulsion force from host polarization and the large charge radius ratio of Zn^2+^ often lead to poor kinetics of Zn^2+^ transport and nonideal intercalation kinetics of Zn^2+^.^[^ ^66^ ^]^ This directly results in low output voltage and low capacity. In addition, the higher desolvation energy of Zn^2+^ at the electrode–electrolyte interface often results in an additional energy penalty for its facile intercalation.^[^ ^67^ ^]^ In contrast, due to their relatively low cost and small migration barrier, the intercalation chemistry of Li^+^/Na^+^ carriers takes place much more easily in host materials, and the smaller polarization of Li^+^/Na^+^ intercalation often exhibits a higher hosting potential. In this case, the (de)intercalation of Li^+^/Na^+^ experiences faster reaction kinetics and more carriers than Zn^2+^ (de)intercalation, which is the basis for achieving a higher output voltage. In this circumstance, the exploration of zinc‐hybrid ESS based on Li^+^ as a carrier has attracted a lot of research interest taking into account the abundance of cathode materials for lithium‐ion battery (LIBs). Therefore, the intercalation/deintercalation of Li^+^ ions, instead of Zn^2+^ ions, repeatedly takes place during the course of charge/discharge. However, the Li^+^ ion intercalation/deintercalation potential of most cathodes for LIBs in aqueous solution exceeds the ESW of conventional aqueous electrolytes. For example, the Li^+^ intercalation potential of a LiVPO_4_F (LVPF) cathode is around 1.45 V (vs saturated calomel electrode (SCE)),^[^ ^68^ ^]^ which is higher than the ESW of conventional aqueous Zn^2+^‐based‐electrolytes, such as ZnSO_4_ and ZnCl_2_. Therefore, proper electrolyte formulation is very important for the construction of high‐voltage hybrid AZBs by this strategy. Recently, highly concentrated dual‐ion electrolytes (HCE) and “water‐in‐salt” electrolytes^[^ ^19^, ^69^, ^70^ ^]^ have been demonstrated to be capable of expanding the working potential of redox electrodes. For instance, Zhi and co‐workers designed an aqueous HCE for the assembly of a high‐voltage zinc hybrid battery consisiting of a Zn anode and an LVPF cathode, as shown in **Figure** **8**a.^[^ ^71^ ^]^ In this case, the zinc hybrid battery exhibited very good reversibility (Figure 8b) due to reversible Zn^2+^ stripping/plating at the anode and Li^+^ intercalation/deintercalation at the cathode. Furthermore, benefitting from the specific feature of the HCE electrolyte, this device delivered an output voltage of ≈1.8 V, which is higher than that of most reported AZBs (Figure 8c). The enhanced voltage is contributed by the large potential difference between the reversible Li^+^ intercalation/deintercalation at the cathode (1.04 V vs SHE) and zinc stripping/plating at the anode (−0.76 V vs SHE). Moreover, zinc‐hybrid batteries based on dual electrolytes have also been developed to attain high‐voltage characteristics. Very recently, Duan and co‐workers established a zinc‐hybrid battery consisting of a Zn–LiMn_2_O_4_ electrode and LiOH–LiNO_3_ hybrid electrolyte (Figure 8d).^[^ ^72^ ^]^ By choosing an alkaline electrolyte and a neutral electrolyte with a cation‐exchange membrane between the two, the ESW of this zinc‐hybrid battery could be extended to 3 V, thus achieving a output voltage up to 2.31 V (Figure 8e) and excellent cycling durability (Figure 8f).

Moreover, high‐voltage Zn‐hybrid batteries with Na^+^ or Mg^2+^ as carriers have also been designed. For example, a Na_3_V_2_O_2*x*_(PO_4_)_2_F_3−2*x*_/CNT cathode for an aqueous sodium ion battery (ASIB) was used to pair with a Zn anode to assembly a hybrid battery.^[^ ^73^ ^]^ This hybrid battery by using Na^+^ as carrier exhibited a high output voltage of 1.65 V (vs Zn/Zn^2+^), which is higher than that of its ASIB counterpart (1.42 V vs Zn/Zn^2+^). In addition, a zinc–magnesium hybrid battery with a MgMn_2_O_4_ cathode affording Mg^2+^ intercalation/deintercalation and a Zn anode (Zn stripping/plating) was also demontrated, exhibiting a tilting plateau at 1.32 V (vs Zn/Zn^2+^).^[^ ^74^ ^]^

### 3.4 Design of Decoupled Systems

Design of a decoupled battery system is another feasible way to achieve high output voltage, which can take advantage of the high redox potential of the cathode and low redox potential of the anode. In general, the decoupled batteries are mainly referred to decouple the working conditions of cathode and anode so as to enable the redox reactions of cathode and anode can be happened simultaneously in a single cell. In such a design, the cathodic electrolyte and anodic electrolyte were seperated by a selective ion‐exchange membrane to avoid mixing. According to the Nernst equation, the potential of the OER (*E*_OER_ *)* and HER (*E*_HER_) can be thus expressed as follows when other substances are in their thermodynamic standard state

(12) $$E_{\text{HER}}=-0.059{\text{ V pH}}_{\text{HER}}$$

(13) $$E_{\text{OER}}=1.23V-0.059V{\text{pH}}_{\text{OER}}$$

Therefore, the ESW of the decoupled aqueous solution can be shown as follows

(14) $$E_{\text{ESW}}=1.23V-0.059V{\text{pH}}_{\text{OER}}+0.059V{\text{pH}}_{\text{HER}}$$

It is obvious that the ESW of the decoupled electrolytes can be broadened using an alkaline electrolyte as anolyte and acidic electrolyte as catholyte. As mentioned before, Zn metal has a lower redox potential (−1.22 V vs SHE) in alkaline solution than in a neutral/acidic electrolyte (−0.76 V vs SHE). On the cathode side, materials with high redox potential in acidic solution could be chosen as far as possible within the oxygen evolution potential.

Recently, the development of decoupled batteries has aroused widespread interest and great progress has been made in increasing the output voltage, especially for decoupled Zn//MnO_2_ batteries. For example, decoupled Zn//MnO_2_ by adopting mild 2 m ZnSO_4_ + 0.1 m MnSO_4_ and alkaline 1 m NaOH + 0.01 m Zn(AC)_2_ as electrolyte with a Na^+^‐form Nafion membrane in between exhibited an average output voltage of up to 1.7 V, which is much higher than that of traditional Zn//MnO_2_ batteries (1.4 V).^[^ ^75^ ^]^ Similarly, Yadav et al. developed decoupled Zn//MnO_2_ aqueous batteries with two electrolytes separated by traditional cellulose separators.^[^ ^76^ ^]^ The MnO_2_ cathode was placed in a Mn^2+^ or ${\text{MnO}}_{4}^{-}$ acidic electrolyte, and the Zn anode was immersed in a polymerized gelled alkaline electrolyte. As a result, this decoupled Zn//MnO_2_ displayed an open circuit potential (OCP) at 2.45 and 2.8 V, respectively, much higher than that of the conventional lead‐acid battery even though the cycling performance was not satisfactory.

**Figure 9:**
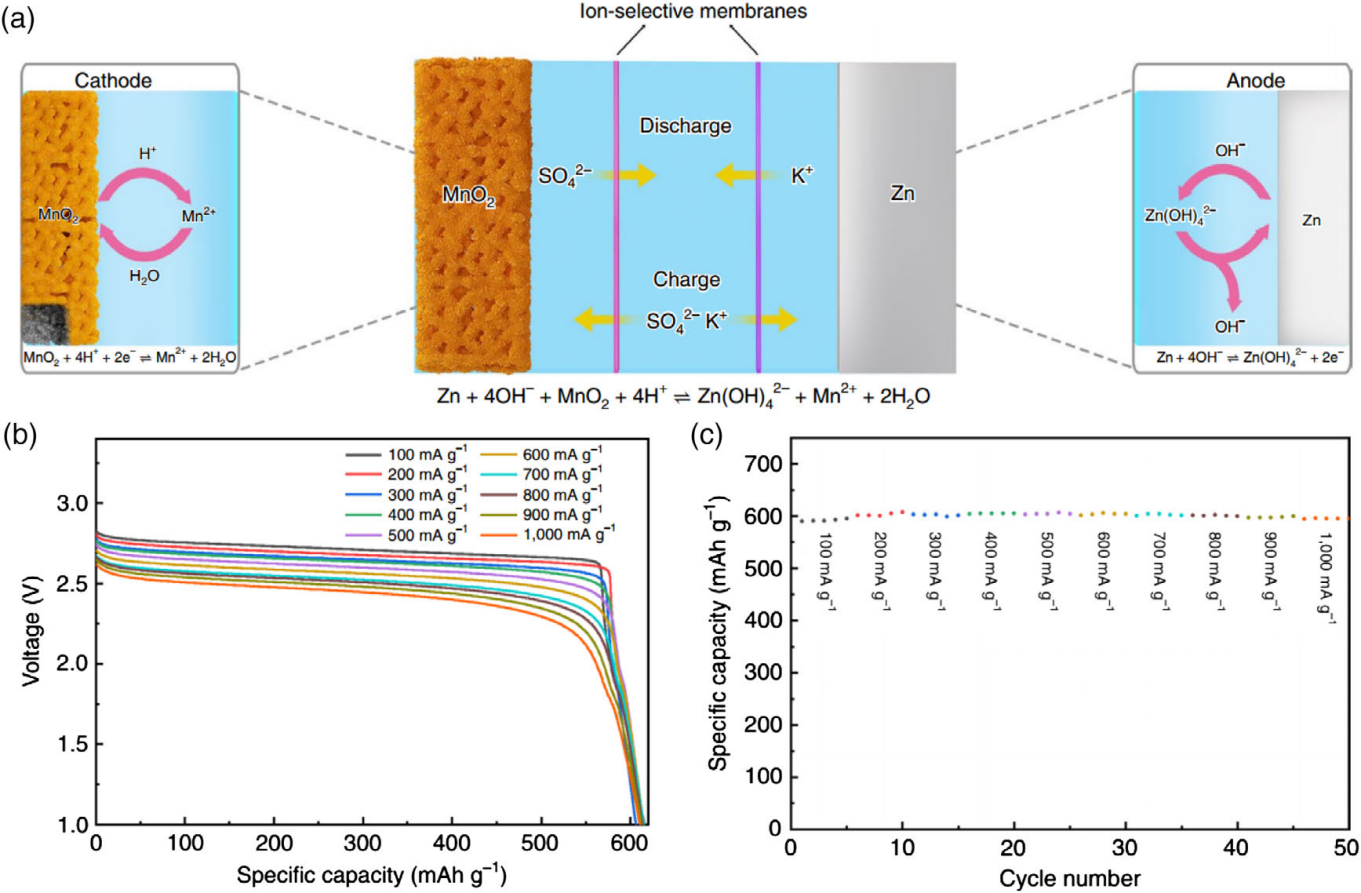
a) Diagram of the decoupled Zn//MnO_2_ battery structure, b) discharge curves, and c) capacity rentation for the decoupled Zn–MnO_2_ battery at different discharging current densities. a–c) Reproduced with permission.^[^ ^77^ ^]^ Copyright 2020, Springer Nature.

Moreover, the utilization efficacy of the MnO_2_ cathode and rate properties of the high‐voltage Zn//MnO_2_ constructed by this strategy should be considered. The redox reaction of MnO_2_ ↔ Mn^2+^ in acid solution gives an ultrahigh theoretical capacity of 616 mAh g^−1^, whereas the most state‐of‐the‐art AZBs affording MnO_2_ ↔ Mn^2+^ transformation only possess a discharge capacity that is below 350 mAh g^−1^. To solve this problem, Hu and co‐workers proposed a novel cell configuration referring to previous works.^[^ ^77^ ^]^ As shown in **Figure** **9**a, the Zn anode and MnO_2_ cathode were separated in two chambers with alkaline and acidic electrolytes, respectively. Smartly, instead of using a simple separator, they designed a central chamber with a neutral electrolyte for ion exchange. Upon discharging, the MnO_2_ is reduced to Mn^2+^ ions dissolved in the electrolyte. In the following charge process, the MnO_2_ is electrodeposited on carbon felt and Zn. This design decouples the Zn//MnO_2_ battery, which enables the two‐electron reaction of MnO_2_/Mn^2+^ (Equation (14)) and makes full use of the loaded MnO_2_, providing a specific capacity of nearly 100% of the theoretical value of 616 mAh g^−1^ (Figure 9b,c) and an output voltage of up to 2.5 V (vs Zn/Zn^2+^). The decoupled electrochemical reactions are
(15)

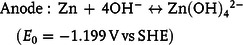



(16)

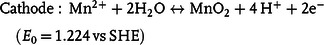




**Figure 10:**
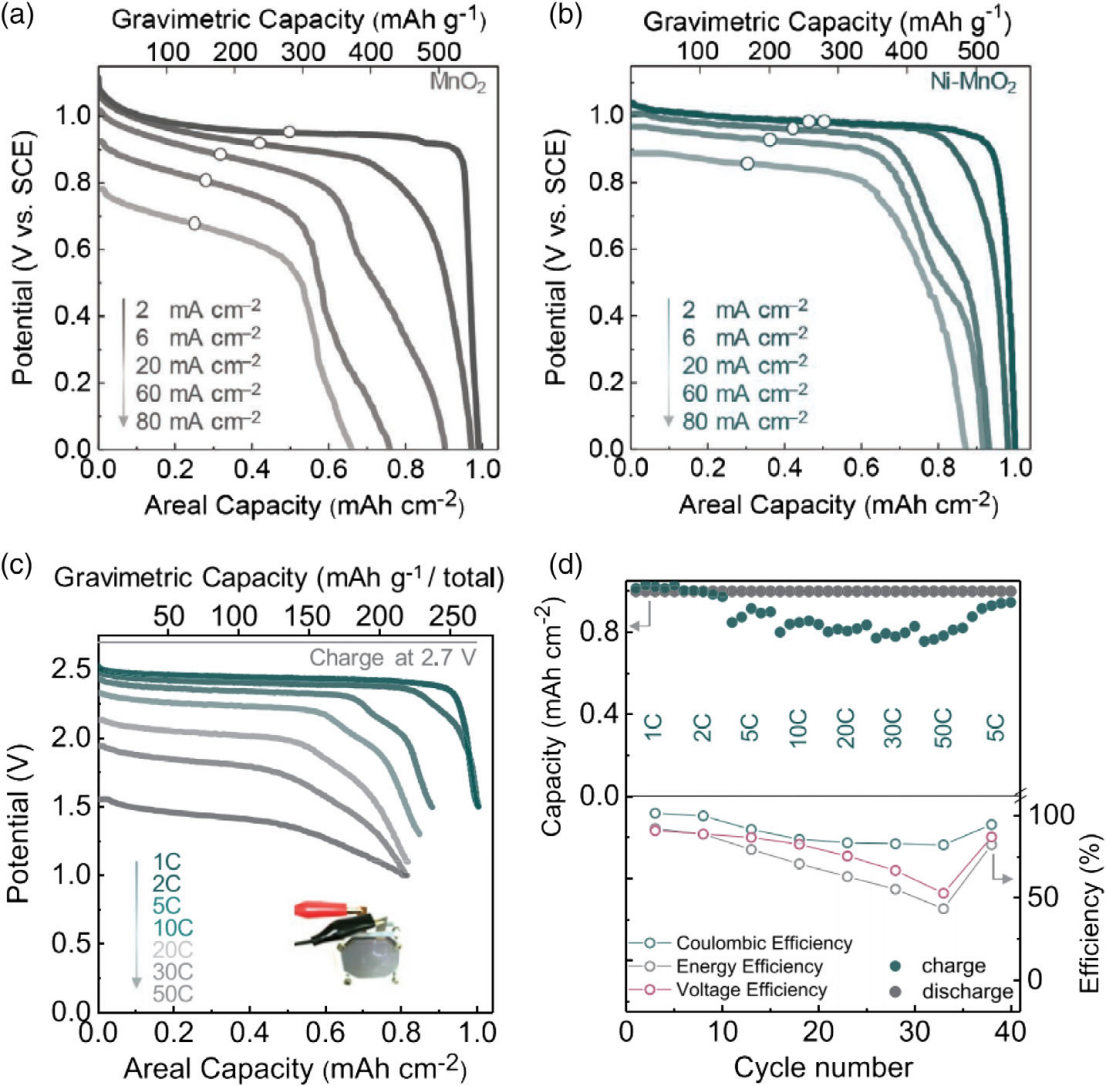
Discharge curves for MnO_2_/Mn^2+^ electrooxidation/electroreduction a) without and b) with a Ni^2+^‐based solution. c,d) The electrochemical performance of the Zn–Mn battery at various current densities from 1 to 50 C. a–d) Reproduced with permission.^[^ ^78^ ^]^ Copyright 2020, Wiley‐VCH.

To obtain affordability in the decoupled Zn//MnO_2_ with stable energy output, the Ni^2+^‐catalyzed electrooxidation/electroreduction method was used to construct a decoupled Zn//MnO_2_ battery.^[^ ^78^ ^]^ In this case, the existence of Ni dopants in MnO_2_ improved the active O 2*p* electron states to facilitate charge transfer, which is beneficial for the electrooxidation/electroreduction of MnO_2_ with rapid electrolysis kinetics. As shown in **Figure** **10**a,b, compared with MnO_2_, Ni–MnO_2_ displays an ultraflat output voltage at 0.99 V (vs SCE) at a current density of 6 mA cm^−2^ and no noticeable potential drops were observed even at the current density of 80 mA cm^−2^. The ultraflat output characteristics are related to the two‐electron redox reaction of MnO_2_/Mn^2+^. It is worth noting that the reversible Zn^2+^ ion insertion/extraction or the successive uptake/removal of both H^+^ and Zn^2+^ in/from bulk materials usually exhibits an inclined discharge curve,^[^ ^4^, ^79^, ^80^, ^81^ ^]^ which is not conducive to achieving a stable energy output. The ultraflat output makes it appealing as a cathode in AZBs. As a result, this decoupled Zn–MnO_2_ battery exhibits fast reaction kinetics (50 C, discharge in 60 s) (Figure 10c), high output voltage (2.44 V), high capacity (≈270 mAh g^−1^ at 2 C), and CE of 99.9% (Figure 10d).

It can be concluded that the voltage window of an alkali––acid electrochemical system can be enhanced by tuning the pH of the anolyte and catholyte. In this manner, high‐voltage AZBs could be obtained by pairing a high‐voltage material in acidic solution with an alkaline zinc anode. Apart from the aforementioned decoupled Zn//MnO_2_ battery, also a decoupled Zn//PbO_2_ battery has been designed to elevate the output voltage. The PbO_2_ material in an acid solution holds the merit of a higher redox potential (1.68 vs SHE) than that of most reported cathode materials for AZBs. Very recently, a decoupled Zn//PbO_2_ hybrid battery with a rather high output voltage greater than 2.9 V was demonstrated by Wen and co‐workers.^[^ ^82^ ^]^ The reaction processes in the cathode and anode are shown in Equation (17) and (18). Upon charging, PbSO_4_ was thoroughly converted into PbO_2_; in the discharge process, the PbO_2_ was electroreduced to PbSO_4_, which was verified by the XRD pattern.
(17)

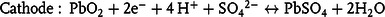



(18)

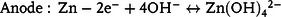




**Figure 11:**
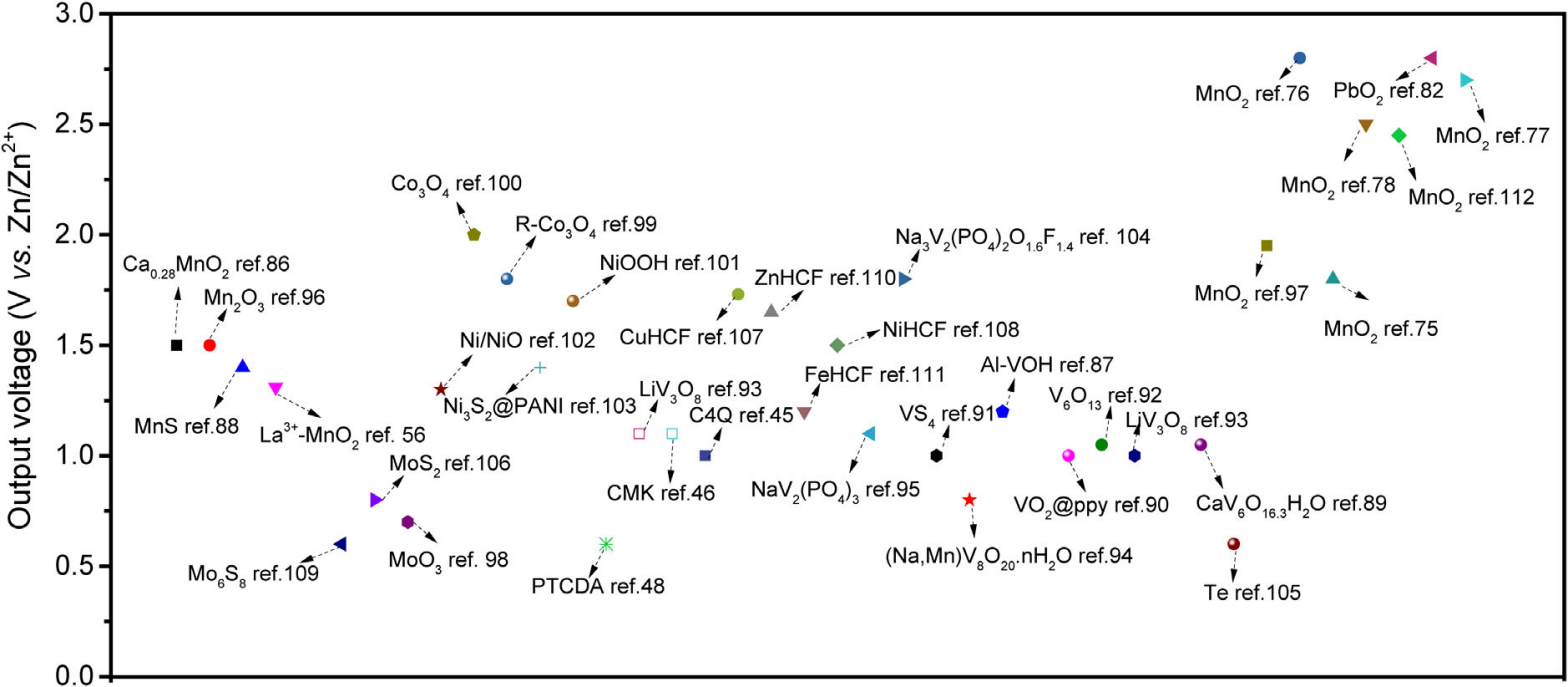
Summary of electrode potentials of reported cathode materials for AZBs.^[^ ^34^, ^45^, ^46^, ^48^, ^56^, ^75^, ^76^, ^77^, ^78^, ^86^, ^87^, ^88^, ^89^, ^90^, ^91^, ^92^, ^93^, ^94^, ^95^, ^96^, ^97^, ^98^, ^99^, ^100^, ^101^, ^102^, ^103^, ^104^, ^105^, ^106^, ^107^, ^108^, ^109^, ^110^, ^111^, ^112^ ^]^

Given these facts, there is still plenty of scope to design multiple electrolytes to construct high‐voltage aqueous batteries. As more cathodes are being discovered, the design of multiple electrolytes that yield wider ESWs could effectively enhance the energy density of AZBs. Although the electrolyte salt used is still relatively cheap, the ceramic membrane or bipolar membrane remains expensive.^[^ ^83^, ^84^ ^]^ Therefore, if we can widen the ESW of the electrolyte to 3.5 V or more and simultaneously use a cheaper membrane, this strategy will be more promising. **Figure** **11** summarizes the output voltage ranges of some recently reported cathode materials. As revealed, most reported AZBs deliver an output voltage that is less than 2.0 V.

## 4 Conclusion

In this minireview, we summarize recently reported strategies for constructing high‐voltage AZBs. The narrow ESW of water (≈1.23 V), imposed by the evolution of hydrogen and oxygen, restricts the output voltage of salt‐in‐water‐based AZBs to below 2 V. Thus, the aqueous batteries usually provide a lower energy density than batteries based on organic electrolytes. To break this problem, various methods have been developed to enhance the output voltage of AZBs, including selection of an appropritate redox potential of the cathode, tunning the redox potential of the cathode, and construction of a decoupled system. Although the output voltage of the state‐of‐the‐art AZBs has been increased to nearly 2.5 V, there are still many challenging issues that need to be solved. 1) Key to building high‐voltage batteries is exploring suitable cathode materials with high redox potential, and redox reactions such as ${\text{MnO}}_{4}^{-}$/MnO_2_ (1.7 V vs SHE) and Ce^4+^/Ce^3+^ (1.28 V vs SHE in 1 m HCl) hold great potential for high‐voltage cathodes for realizing their reversibility. In addition, the redox couple NiOOH/NiO achieves a higher potential in a neutral electrolyte than in an alkline one, which makes the neutral aqueous Ni//Zn battery expected to boost the output voltage to ≈1.8 V. However, the redox potential of these materials is beyond the ESW of the aqueous solution; thus, it is challenging to apply them to AZBs. Searching for aqueous electrolytes with a wide ESW (>3 V) is an important prerequisite for achieving high‐voltage AZBs. Highly concentrated aqueous electrolytes, such as WISE, were proposed as promising electrolytes that exhibit a high ESW of ≈3.0 V, and have been successfully applied to high‐voltage aqueous lithium‐ion batteries. Therefore, with the increasing understanding of the reaction mechanism of LIBs in WISE, it provides a good starting platform for the development of a new WISE system for high‐voltage AZBs. 2) Although a large number of cathode materials have been reported, great efforts are still required to develop cathode materials that possesses high output voltage. Structural engineering, such as neutral molecule intercalation (e.g., H_2_O) or ion doping (e.g., Co^2+^, Fe^2+^), could change the crystal structure and electronic structure of the material, thus effectively improving the redox potenital of cathode materials. However, the intercalation site and amount will play an importance role in further optimizations. Due to the existence of electrostatic repulsion, a cation‐doped material may have a strong repulsion to Zn^2+^ ions, thereby reducing the reversibility of Zn^2+^ insertion/extraction, whereas anion‐doped materials have the opposite effect. Thus, cointercalation of anions with electroneutral molecules (e.g., aeteropoly acid, organic macromolecule) in AZB cathodes to enhance the output voltage of AZBs is still a topic deserving more research. How to control the intercation/doping sites and amounts accurately, such as by the template method or by oriented synthesis, is also a promising topic. 3) The conventional working mechanism of AZBs is the insertion/extraction of Zn^2+^ or mixed Zn^2+^/H_3_O^+^ in the cathode materials. However, Zn^2+^ owns a high charge, large hydrate ion radius, and large desolvation energy, which lead to the Zn^2+^ intercalation chemistry exhibiting tardy kinetics and poor cycling performance. The large diffusion energy barrier caused by the strong repulsive force not only results in structure distortion, but also in large polarization, leaving an unsatisfactory output voltage and rate capability. Compared with Zn^2+^, some low‐charge carriers such as Li^+^, Na^+^, and H_3_O^+^ are promising due to their small ionic size (≈1.0 Å). Therefore, an alternative approach for building high‐voltage and high‐rate AZBs is to develop new cathodes with desirable crystal structure that can use Li^+^, Na^+^, or H_3_O^+^ as charge carriers for the host material reaction. Some high‐voltage cathode materials for lithium‐ and sodium‐ion batteries can be used to construct high‐voltage AZBs, such as layered LiCoO_2_ and layered Na_*x*_MO_2_ oxides. It should be noted that Li^+^ or Na^+^ intercalation cathodes are not necessarily the best choice. Coinsertion/extraction of dual carriers (Li^+^ and Na^+^) or more into the host materials during the charge/discharge process may improve the operating voltage of the battery because of the simultaneous insertion of dual or more carriers into the host materials, which would enhance the synergetic effect of their insertion kinetics and thermodynamics. In addition, the anion insertion chemistry also should be considered to improve the operating voltage of the battery. Moreover, building a water chain in layer/tunnel‐structured materials (e.g., CuHCF, α‐MoO_3_, MoS_2_, and δ‐MnO_2_) blocking Zn^2+^ transport and utilizing the Grotthuss mechanism may achieve selective H_3_O^+^ intercalation in a neutral electrolyte.^[^ ^85^ ^]^ 4) Cost and stable performance should be considered when designing electrolyte and electrode materials for high‐voltage AZBs. Although the satisfactory discharge voltage of ≈2.5 V has been obtained in a decoupled Zn//MnO_2_ system, expensive membranes were used. Therefore, if we can use a cheaper membrane (e.g., ceramic membrane, fiberglass membrane), these decoupled batteries will hopefully be commercialized. The cheaper membrane needs to be designed with a high ion exchange rate, good interfacial contact with the electrolyte, and wide electrochemical and thermal stability windows. For example, the surface of an inexpensive, readily available fiberglass membrane could be modified by coating both sides with metal oxides or an oganic polymer to block the mixing of H^+^ and OH^−^ and allow other ions to pass through. In addition, low rate capability and poor cycling stability are the two other main barriers that impede the application of decoupled batteries. Therefore, design of a new electroplating battery system in a single electrolyte may also be applicable for improving the rate capability and cycling performance of high‐voltage AZBs.

## Conflict of Interest

The authors declare no conflict of interest.
